# Increased Libido Following Brivaracetam Initiation: A Case Report of Temporal Lobe Epilepsy

**DOI:** 10.1002/npr2.70160

**Published:** 2026-08-31

**Authors:** Toru Horinouchi, Taiki Matsuyama, Mayusa Mito, Yuichi Nakamura, Takahiro A. Kato

**Affiliations:** ^1^ Department of Psychiatry Hokkaido University Graduate School of Medicine Sapporo Hokkaido Japan; ^2^ Department of Psychiatry and Neurology Hokkaido University Hospital Sapporo Hokkaido Japan; ^3^ Hokkaido University Epilepsy Center Sapporo Hokkaido Japan; ^4^ Department of Psychiatry Wakkanai City Hospital Wakkanai Hokkaido Japan

**Keywords:** brivaracetam, dopamine D2 receptor, Klüver–Bucy syndrome, libido, temporal lobe epilepsy

## Abstract

**Background:**

Sexual dysfunction is a common comorbidity in patients with epilepsy; it correlates with seizure frequency and right temporal lobe epilepsy and affects quality of life (QOL). Despite its clinical significance, sexual dysfunction is often underassessed in clinical practice. Brivaracetam (BRV), an anti‐seizure medication (ASM) introduced in recent years, is considered to have few psychiatric side effects, and no association with sexual dysfunction has been reported to date. We report a case of temporal lobe epilepsy in which the patient exhibited increased libido following the initiation of BRV.

**Case Presentation:**

A woman in her 70s with drug‐resistant temporal lobe epilepsy, weekly focal seizures, and mild cognitive impairment was treated with brexpiprazole (BXP) for delusions of theft. BRV (50 mg/day) was initiated due to worsening epileptic seizures. Three weeks later, she developed persistent, intrusive sexual urges accompanied by self‐stimulatory behavior (e.g., genital touching). She initially concealed these symptoms due to embarrassment but disclosed them at the follow‐up 1 month later. Given the temporal association with BRV initiation, the drug was discontinued, resulting in marked improvement within 2 weeks and subsequent resolution. Throughout the course of treatment, no changes were observed in seizure frequency, cognitive function, or other ASMs.

**Discussion:**

Previous reports have described increased libido with levetiracetam, which shares the same mechanism of action with BRV. Genetic factors, such as reduced striatal dopamine receptor density associated with DRD2/ANKK1 polymorphisms may contribute to psychiatric side effects. In this case, BRV‐associated modulation of neurotransmission may have interacted with a vulnerable dopaminergic system, as indicated by the need for BXP. Preexisting bilateral hippocampal atrophy may also have provided a structural substrate resembling a pharmacologically induced incomplete Klüver–Bucy syndrome. Clinicians should be aware that patients may hesitate to report such symptoms and should actively monitor for changes in libido after initiating BRV.

## Abstract 2

This case report highlights a patient with temporal lobe epilepsy who experienced a distressing libido increase following brivaracetam initiation. Although primarily associated with brivaracetam, incomplete Klüver–Bucy syndrome and concurrent brexpiprazole may have also been involved.

## Graphical abstract

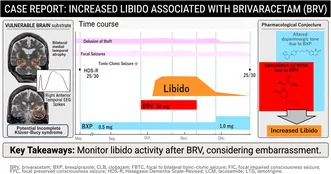

## 1 Background

Sexual dysfunction in epilepsy occurs in 20%–78% of women and 17.5%–79% of men [1]. It correlates with seizure frequency [2], is more common in right temporal lobe epilepsy [3], and negatively affects quality of life (QOL) [4]. Although hyposexuality is more typical, hypersexuality has been reported in 14% of patients after temporal lobe epilepsy surgery [5]. Despite its clinical importance, sexual dysfunction is often underrecognized in practice [6].

Brivaracetam (BRV), which has been introduced in recent years as a new treatment option, is an anti‐seizure medication (ASM) that selectively binds to synaptic vesicle protein 2A (SV2A) at nerve terminals. In Phase III trials of BRV, the main reported side effects included drowsiness, dizziness, headache, and fatigue [7]; however, no adverse events related to sexual function or libido were observed.

We report a case in which a patient with temporal lobe epilepsy complained of a marked increase in libido following the initiation of BRV. To the best of our knowledge, this is the first case report of a libido increase associated with BRV.

## 2 Case Presentation

Written informed consent was obtained from the patient for publication.

The patient was right‐handed and had two siblings. Her development was normal, with no history of febrile seizures. After graduating from junior high school, she worked as a hairdresser for over 10 years. Following the onset of epilepsy, she left her job and lived with her parents and younger brother. In her 50s, she moved to a group home, where she currently resides and attends a day service program twice weekly.

She was diagnosed with temporal lobe epilepsy following an initial focal impaired consciousness seizure (FIC) with oroalimentary automatisms at age 28. She later began experiencing tonic–clonic seizures. She was referred to our department in her 40s and has been under our care since then. She had been hospitalized multiple times, but her seizures were drug‐resistant. Although receiving clobazam (CLB), lamotrigine (LTG), and lacosamide (LCM), she experienced three seizure types (ILAE 2025 classification) [8]: weekly focal preserved consciousness seizures (FPC) with numbness or a being pulled sensation in the head; monthly focal impaired consciousness seizures (FIC) without aura, sometimes causing falls; and annual focal‐to‐bilateral tonic–clonic seizures (FBTC).

**FIGURE 1:**
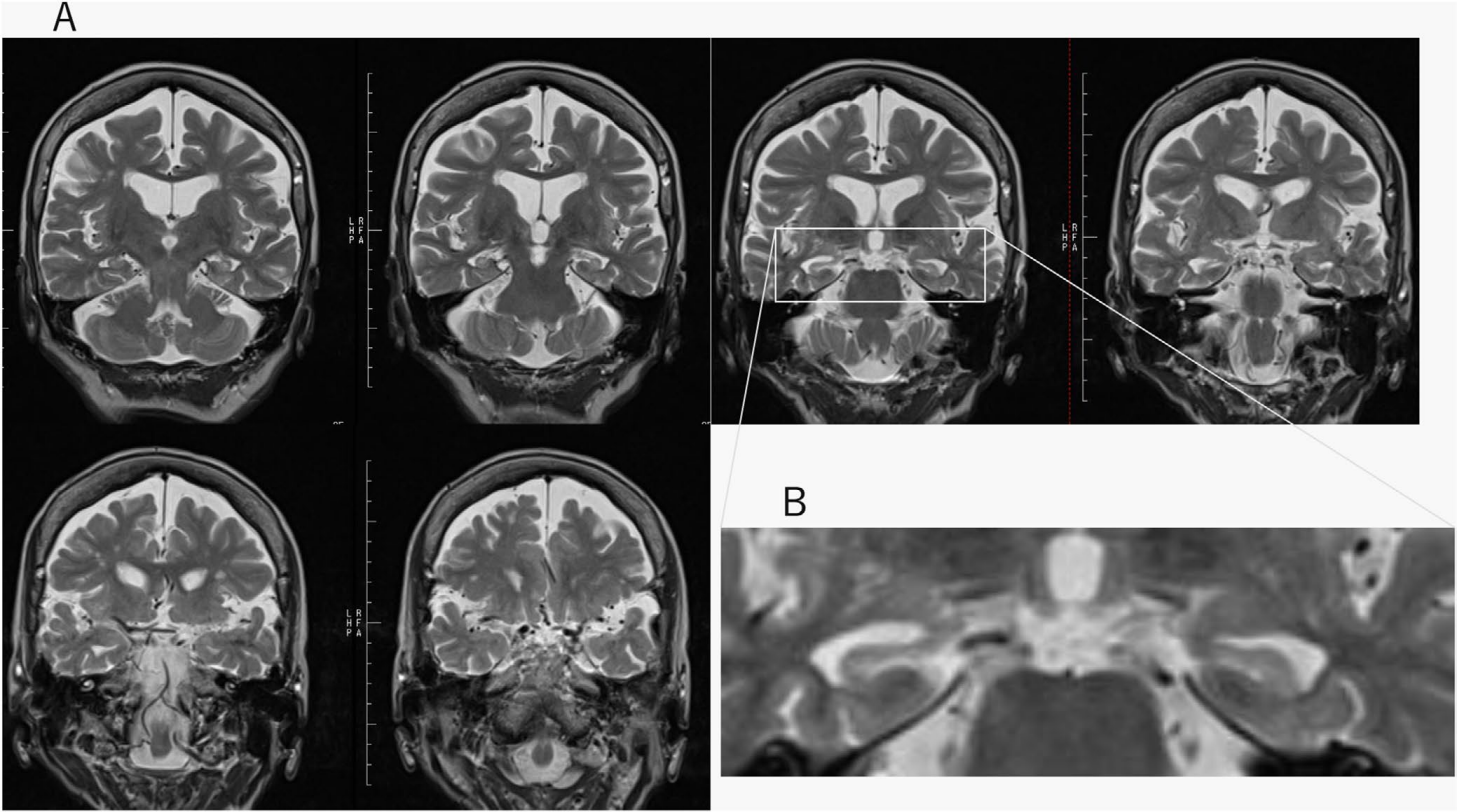
Shows a coronal T2‐weighted image. Mild generalized atrophy is detected (A). There is bilateral medial temporal atrophy including hippocampi and amygdalae, with a slight right‐sided predominance, and blurring of the layered structure of the hippocampus is noted (B). Similar findings were also observed on an MRI taken 20 years ago.

**FIGURE 2:**
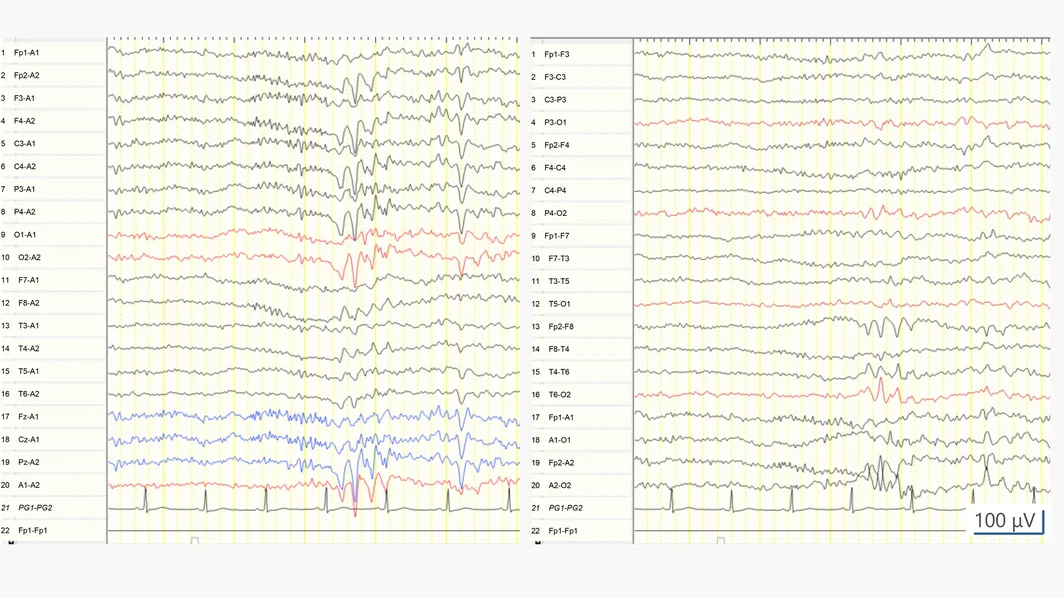
Shows electroencephalogram (EEG) findings for the reference montage (A) and bipolar montage (B). A spike‐and‐wave centered in the right anterior temporal region (F8 and T4) is observed. The recording parameters for both EEGs are a sensitivity of 10 μV/mm, a time constant of 0.3 s, and a high‐cut filter of 120 Hz.

In her 70s, she began complaining of forgetfulness. She scored 24 out of 30 on the Hasegawa Dementia Scale‐Revised (HDS‐R), indicating mild cognitive impairment. One year later, delusions of theft and associated displays of anger began. MRI revealed mild bilateral hippocampal atrophy with a slight predominance on the right, as well as blurring of the hippocampal layer structure (Figure 1). Electroencephalography (EEG) also revealed spike‐and‐wave discharges centered in the right anterior temporal region, consistent with previous findings (Figure 2). To treat these behavioral and psychological symptoms of dementia (BPSD), 0.5 mg brexpiprazole was started in May. In view of a recent FBTC, an adjustment to the ASM was considered during a follow‐up visit, and 50 mg BRV was started in September.

**FIGURE 3:**
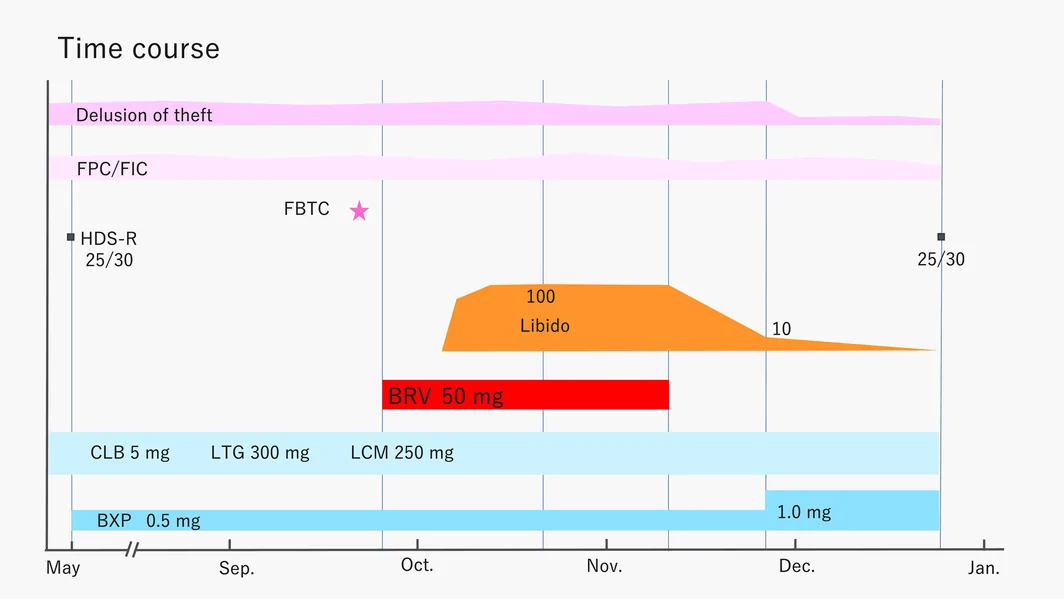
Shows the time course of the patient. The vertical lines indicate hospital visits. BRV, brivaracetam; BXP, brexpiprazole; CLB, clobazam; FBTC, focal‐to‐bilateral tonic–clonic seizure; FIC, focal impaired consciousness seizure; FPC, focal preserved consciousness seizure; HDS‐R, Hasegawa Dementia Scale‐Revised; LCM, lacosamide; LTG, lamotrigine.

Approximately 3 weeks later, she experienced a subjective, profound increase in sexual drive, characterized by persistent sexual fantasies that spontaneously occupied her mind and caused distress. This intense surge in desire was accompanied by genital self‐stimulation to relieve the tension. Although her embarrassment delayed the disclosure of these symptoms until her November consultation—leaving them unreported in October—the suspected temporal association led to the discontinuation of BRV. Two weeks later, the symptoms had subjectively decreased to approximately 10% of their original intensity and subsequently resolved. Throughout the course of treatment, there were no changes in HDS‐R scores, FPC and FIC frequency, or ASM other than BRV. The delusion of theft persisted but improved slightly when the dose of BXP was increased (Figure 3).

## 3 Discussion and Conclusion

This case describes increased libido following BRV initiation.

The extended adverse event profile (E AEP) was developed to systematically capture ASM side effects [9]. It includes sexual function and drive and identifies LEV, valproic acid, and topiramate as risk factors; however, the patient was not receiving these drugs. A MEDLINE search revealed no prior reports linking BRV to libido or sexual dysfunction. A search was subsequently conducted using VigiAccess—a component of the WHO International Drug Monitoring Programme (WHO PIDM) that compiles individual case safety reports from more than 110 countries worldwide—and four reports of increased libido associated with BRV were identified [10]. Although further details are not available through this service, this suggests a possible association between BRV and increased libido.

We next examined the association with LEV, which shares a mechanism of action with BRV. Although rare, increased libido has been reported with LEV [11, 12], suggesting that SV2A ligands may influence sexual drive. Individual biological vulnerability may be involved in the manifestation of such psychiatric side effects. In particular, compared with noncarriers, carriers of the T allele of the dopamine D2 receptor gene (DRD2/ANKK1) have been reported to have lower D2 receptor density in the striatum, and the presence of the T allele has been shown to correlate with the incidence of psychiatric side effects associated with LEV [13]. In the present case, BRV may have modified neurotransmitter release within the reward circuitry, triggering increased libido against a background of dopaminergic dysregulation associated with BXP use. BXP is known to possess a high affinity and partial agonism for both dopamine D2 and D3 receptors [14]. Interestingly, a case of BXP‐associated hypersexuality has been documented, and it has been suggested that this adverse event may be linked to its high affinity and partial agonist profiles at D2 and D3 receptors within the reward system [15]. In our case, the patient did not report any increase in libido during the preceding period of BXP treatment without BRV, indicating that BXP alone cannot fully explain the symptom. However, it cannot be ruled out that the initiation of BRV in the context of altered dopaminergic tone due to BXP's D2/D3 partial agonism contributed to the development of increased libido.

Furthermore, the association between epilepsy and sexual behavioral abnormalities requires further investigation. Patients with temporal lobe epilepsy may present with incomplete periictal Klüver–Bucy syndrome [16]. Klüver–Bucy syndrome was originally discovered in monkeys following bilateral temporal lobectomy, which resulted in behavioral disturbances such as hyperorality, psychic blindness, and altered sexual behavior [17]; however, in patients with epilepsy, symptoms often manifest in an incomplete form rather than presenting all symptoms together [18]. In fact, multiple cases of ictal kissing [19] and excessive masturbation following selective amygdalohippocampectomy [20] have been reported. In this case, bilateral medial temporal atrophy was observed, suggesting the presence of an organic basis that was potentially prone to dysfunction of the limbic network. Given this background, it cannot be ruled out that the initiation of BRV led to an increase in libido.

There are several limitations to this discussion. Our assessment relied entirely on the patient's subjective self‐reporting, and no objective or standardized scales for sexual dysfunction (e.g., Arizona Sexual Experience Scale or Changes in Sexual Functioning Questionnaire) were utilized. Furthermore, because no EEG was performed during the period of increased libido, it remains unclear whether forced normalization contributed to this clinical course, although there was no change in seizure frequency.

This appears to be the first reported case of increased libido associated with BRV. In addition to a potential direct drug effect, contributing factors may include dopaminergic dysregulation related to BXP and temporal lobe epilepsy accompanied by bilateral hippocampal atrophy. Clinicians should actively monitor for psychiatric side effects, including changes in libido, and recognize that patients may be reluctant to report such symptoms.

## Author Contributions

T.H. carried out the examination, diagnosis, and treatment. T.H. wrote the manuscript with support from T.M., M.M., Y.N., and T.A.K. All authors contributed to the final version of the manuscript.

## Funding

The authors have nothing to report.

## Disclosure

The authors have nothing to report.

## Ethics Statement

The authors have nothing to report.

## Consent

Written informed consent was obtained from the patient for publication of this case report and any accompanying images.

## Conflicts of Interest

The authors declare no conflicts of interest.

## Data Availability

The data that support the findings of this case report are not publicly available due to privacy concerns and ethical restrictions related to the protection of patient confidentiality, as the dataset contains potentially identifiable clinical information. Further details may be made available from the corresponding author upon reasonable request, subject to appropriate ethical approval.
